# Primary radiotherapy and deep inferior epigastric perforator flap reconstruction for patients with breast cancer (PRADA): a multicentre, prospective, non-randomised, feasibility study

**DOI:** 10.1016/S1470-2045(22)00145-0

**Published:** 2022-05

**Authors:** Paul T R Thiruchelvam, Daniel R Leff, Amy R Godden, Susan Cleator, Simon H Wood, Anna M Kirby, Navid Jallali, Navita Somaiah, Judith E Hunter, Francis P Henry, Aikaterini Micha, Rachel L O'Connell, Kabir Mohammed, Neill Patani, Melissa L H Tan, Dorothy Gujral, Gillian Ross, Stuart E James, Aadil A Khan, Jennifer E Rusby, Dimitri J Hadjiminas, Fiona A MacNeill, PRADA Trial Management Group, Paul TR Thiruchelvam, Daniel R Leff, Amy R Godden, Susan Cleator, Simon H Wood, Anna M Kirby, Navita Somaiah, Neill Patani, Dorothy Gujral, Gillian Ross, Stuart James, Aadil Khan, Jennifer E Rusby, Dimitri Hadjiminas, Fiona A MacNeill

**Affiliations:** a Department of Breast Surgery, Imperial College Healthcare NHS Trust, London, UK; b Department of Clinical Oncology, Imperial College Healthcare NHS Trust, London, UK; c Department of Plastic and Reconstructive Surgery, Imperial College Healthcare NHS Trust, London, UK; d BioSurgery and Surgical Technology, Department of Surgery, Imperial College London, London, UK; e Department of Breast Surgery, Royal Marsden NHS Foundation Trust, London, UK; f Department of Plastic and Reconstructive Surgery, Royal Marsden NHS Foundation Trust, London, UK; g The Institute of Cancer Research, London, UK; h Department of Breast Surgery, University College London Hospitals NHS Trust, London, UK; i Department of Breast Surgery, Birmingham City Hospital, Birmingham, UK

## Abstract

**Background:**

Radiotherapy before mastectomy and autologous free-flap breast reconstruction can avoid adverse radiation effects on healthy donor tissues and delays to adjuvant radiotherapy. However, evidence for this treatment sequence is sparse. We aimed to explore the feasibility of preoperative radiotherapy followed by skin-sparing mastectomy and deep inferior epigastric perforator (DIEP) flap reconstruction in patients with breast cancer requiring mastectomy.

**Methods:**

We conducted a prospective, non-randomised, feasibility study at two National Health Service trusts in the UK. Eligible patients were women aged older than 18 years with a laboratory diagnosis of primary breast cancer requiring mastectomy and post-mastectomy radiotherapy, who were suitable for DIEP flap reconstruction. Preoperative radiotherapy started 3–4 weeks after neoadjuvant chemotherapy and was delivered to the breast, plus regional nodes as required, at 40 Gy in 15 fractions (over 3 weeks) or 42·72 Gy in 16 fractions (over 3·2 weeks). Adverse skin radiation toxicity was assessed preoperatively using the Radiation Therapy Oncology Group toxicity grading system. Skin-sparing mastectomy and DIEP flap reconstruction were planned for 2–6 weeks after completion of preoperative radiotherapy. The primary endpoint was the proportion of open breast wounds greater than 1 cm width requiring a dressing at 4 weeks after surgery, assessed in all participants. This study is registered with ClinicalTrials.gov, NCT02771938, and is closed to recruitment.

**Findings:**

Between Jan 25, 2016, and Dec 11, 2017, 33 patients were enrolled. At 4 weeks after surgery, four (12·1%, 95% CI 3·4–28·2) of 33 patients had an open breast wound greater than 1 cm. One (3%) patient had confluent moist desquamation (grade 3). There were no serious treatment-related adverse events and no treatment-related deaths.

**Interpretation:**

Preoperative radiotherapy followed by skin-sparing mastectomy and immediate DIEP flap reconstruction is feasible and technically safe, with rates of breast open wounds similar to those reported with post-mastectomy radiotherapy. A randomised trial comparing preoperative radiotherapy with post-mastectomy radiotherapy is required to precisely determine and compare surgical, oncological, and breast reconstruction outcomes, including quality of life.

**Funding:**

Cancer Research UK, National Institute for Health Research.

## Introduction

A longstanding challenge in oncoplastic breast surgery is the optimal integration of breast reconstruction and post-mastectomy radiotherapy, which is compounded by broadening indications for post-mastectomy radiotherapy. Patients increasingly choose immediate breast reconstruction, but post-mastectomy radiotherapy is associated with complications. Compared with non-irradiated implants, irradiated implants have been shown to be associated with a greater risk of infection (relative risk [RR] 2·44), capsule formation (5·47), and reconstruction failure (3·32).^1^ Similarly, irradiated autologous flaps have shown greater rates of fibrosis or shrinkage (RR 35·00),^2^ flap contracture (11·00),^2^ volume loss (8·16),^3^ and fat necrosis (1·91)^3^ than non-irradiated autologous flap reconstruction. Regardless of reconstructive technique, post-mastectomy radiotherapy has been shown to be associated with inferior patient satisfaction.^4^ Furthermore, postoperative complications following immediate reconstruction delays radiotherapy (by a mean of 19·7 days [95% CI 8·78–30·61]),^5^ which might compromise oncological outcomes.^6^

Many surgeons in the UK recommend delaying reconstruction if post-mastectomy radiotherapy is required.^7^ In the UK, 49–55% of microvascular breast reconstructions are delayed, as highlighted in both the UK National Flap Registry^8^ and the Getting It Right First Time quality improvement initiative.^9^ Therefore, some women have still not had breast reconstruction even several years after mastectomy.^10^ Compared with delayed reconstruction, immediate breast reconstruction can protect women from psychosocial distress, negative body image, and diminished sexual wellbeing.^11^ Alternatively, the delayed–immediate pathway (appendix p 1) uses a temporary implant to support the breast skin envelope during post-mastectomy radiotherapy, with subsequent conversion to autologous reconstruction.^12^ However, compared with delayed reconstruction, this pathway is associated with increased risk of peri-implant infection (50% *vs* 10%) and unplanned explantation.^13^

**Research in context**

**Evidence before this study**

We searched MEDLINE and PubMed for studies published from database inception to Jan 1, 2015, for papers published in English, using the terms “breast reconstruction”, “neoadjuvant”, “preoperative”, “radiotherapy”, “preoperative radiotherapy”, and “breast cancer”. Two authors (PTRT and DRL) screened relevant abstracts and full-text articles to obtain background information on the topic. We did not apply a formal screening process for included and excluded articles and documents. Preoperative radiotherapy studies in breast cancer treatment were mostly retrospective and did not report on treatment timelines. These studies compared preoperative radiotherapy with chemoradiotherapy (rather than compared with post-mastectomy radiotherapy) and many did not report on oncological outcomes using survival analyses. We identified seven studies of preoperative radiotherapy and breast reconstruction, but none reported on contemporary microvascular reconstruction. This study aimed to address major knowledge gaps about the safety of preoperative radiotherapy followed by skin-sparing mastectomy and autologous microsurgical reconstruction in patients with breast cancer requiring mastectomy.

**Added value of this study**

To our knowledge, this is the first multicentre, prospective, non-randomised, feasibility study to provide comprehensive data on the safety and aesthetic outcome of preoperative radiotherapy in patients with breast cancer who had skin-sparing mastectomy and deep inferior epigastric perforator (DIEP) flap reconstruction. This study shows that autologous free-flap surgery is feasible and technically safe if performed within 6 weeks of completion of preoperative radiotherapy. In addition, preoperative radiotherapy can reduce treatment pathway delays that occur with post-mastectomy radiotherapy due to wound healing issues. Consequently, clarification of the feasibility and safety of complex surgery after preoperative radiotherapy, combined with the potential to reduce overall treatment time, might encourage multidisciplinary teams (tumour boards) who are concerned about adverse effects of post-mastectomy radiotherapy on breast reconstruction to consider immediate breast reconstruction.

**Implications of the available evidence**

Evidence to support preoperative radiotherapy and immediate breast reconstruction is mainly historical, and there is little evidence for contemporary DIEP flap reconstruction. This study shows that skin-sparing mastectomy and immediate microvascular breast reconstruction can be safely performed after preoperative radiotherapy, with low rates of postoperative complications and good aesthetic results. Further research is now required to compare surgical, oncological, and quality-of-life outcomes of breast reconstruction in a randomised trial of preoperative radiotherapy compared with conventional post-mastectomy radiotherapy.

Given the limitations of these pathways, interest has grown in preoperative radiotherapy to avoid the adverse effects of radiation on autologous breast reconstruction. Additionally, preoperative radiotherapy might streamline treatment by minimising delays associated with adjuvant post-mastectomy radiotherapy.^5^ Preoperative radiotherapy might also achieve an antitumour response eradicating subclinical disease, and potentially increase rates of pathological complete response through the radiosensitising effects of neoadjuvant chemotherapy.^14^ However, there is a paucity of data on the role of preoperative radiotherapy before mastectomy and microvascular autologous reconstruction.^15^ Studies reporting on the safety of preoperative radiotherapy with mastectomy and reconstruction have largely involved pedicle flaps with or without implants,^14^, ^16^, ^17^, ^18^ and only one study reported on microvascular reconstruction.^15^ Of note, that study included patients with inflammatory breast cancer and skin involvement, half (49%) of whom did not have skin-sparing mastectomy, more than half (52%) had a scheduled reoperation for delayed flap inset, and not all patients received microsurgical reconstruction. We aimed to evaluate the feasibility of preoperative radiotherapy followed by skin-sparing mastectomy with immediate deep inferior epigastric perforator (DIEP) flap reconstruction in patients with breast cancer requiring mastectomy.

## Methods

### Study design and participants

We conducted a prospective, non-randomised, feasibility study (PRADA) at two National Health Service (NHS) trusts in the UK (Royal Marsden NHS Foundation Trust, London, and Imperial College Healthcare NHS Trust, London). Eligible patients were women aged older than 18 years with a laboratory diagnosis of primary breast cancer requiring mastectomy either for extensive disease or positive margins following breast-conserving surgery, who were suitable for DIEP flap reconstruction and were predicted to need post-mastectomy radiotherapy (appendix p 2). A full list of inclusion and exclusion criteria is shown in the protocol (appendix). Patients were mainly identified at diagnosis when the multidisciplinary team recommended neoadjuvant chemotherapy and mastectomy, and post-mastectomy radiotherapy was expected to be required (appendix p 2). Patients who were not candidates for breast-conserving surgery due to inadequate response to chemotherapy were also eligible. Patients were counselled by a radiation oncologist and breast and plastic surgeon regarding the novel treatment sequence.

Written informed consent was obtained before patients received preoperative radiotherapy and surgery. Local regional ethics committee approval was obtained from the Camden and Kings Cross Research Ethics Committee (15/LO/1071). The study was registered with ClinicalTrials.gov 5 months after recruitment began, during which time two patients were recruited.

### Procedures

Baseline demographic data were obtained for age, ethnicity, body-mass index, smoking status, and history of diabetes. Tumour subtype and grade were recorded. Tumour receptor status was defined as either positive or negative on the basis of oestrogen receptor status (Allred score ≥3 out of 8 was defined as positive), progesterone receptor status (Allred score ≥3 was defined as positive), and human epidermal growth factor receptor (HER2) status (immunohistochemical stain ≥3 or fluorescent in-situ hybridisation-positive defined as positive). Stage was recorded according to the American Joint Committee on Cancer.^19^

**Figure 1:**
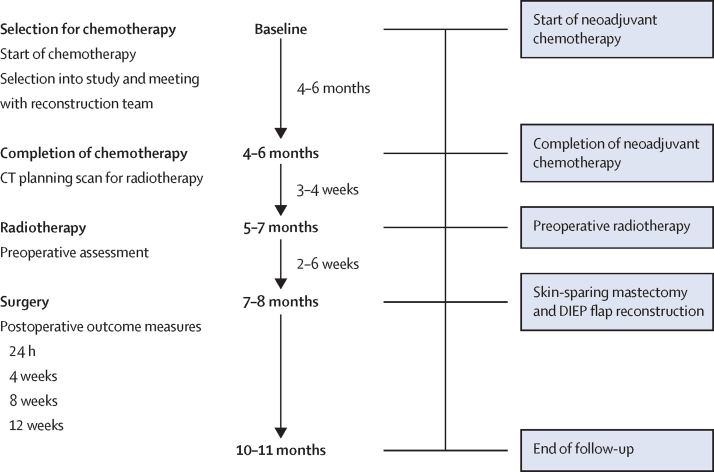
Study plan and treatment schedule DIEP=deep inferior epigastric perforator.

Preoperative radiotherapy was planned to commence 3–4 weeks after neoadjuvant chemotherapy (figure 1). Radiological monitoring of response with breast and axillary ultrasonography and MRI was done as per each trust's standard care. Dose reductions or modifications were as per clinical need and were not prespecified in the protocol. Skin-sparing mastectomy and DIEP flap reconstruction were planned for 2–6 weeks after completing preoperative radiotherapy, on the basis that a reduced complication profile had been shown if surgery was expedited (<6 weeks).^20^ Dates of treatment commencement and completion were captured prospectively.

At both sites, radiotherapy delivery followed the London Cancer Alliance Breast Cancer Clinical Guidelines. All patients had a radiotherapy-planning CT scan in a standard semi-supine position and, when indicated, using a breath-holding technique. Radiotherapy CT scan slices were acquired at no greater than 5 mm intervals, but ideally at 3 mm intervals. The clinical target volume included the breast tissue (with or without skin) with or without the lymph node basins, including the axilla (level I and II), supraclavicular fossa (level III and IV), and internal mammary nodes. Before radiotherapy planning, the multidisciplinary team defined the extent of planned axillary surgery and the need for radiotherapy. For example, in those planned to have an axillary clearance and who required radiotherapy to the supraclavicular fossa, the nodal volume extended from the upper extent of planned surgery to include the supraclavicular fossa (levels III and IV). The internal mammary node chain was included in stage N2–3 disease or if preoperative involvement was identified on staging PET–CT scans. Target volumes (breast and nodal clinical target volumes) were defined as per European Society for Radiotherapy and Oncology consensus guidelines.^21^ Nodal clinical target volumes were expanded by 5 mm to produce planning target volumes. Organs-at-risk were defined as per standard practice. Study centres were permitted to apply local protocols with respect to skin build-up. At the Royal Marsden NHS Foundation Trust, a 5 mm wax bolus was applied to the skin for all fractions on five patients with stage T4 disease. At the Imperial College Healthcare NHS Trust, a 10 mm bolus was applied for half the fractions in all patients.

Treatment plans aimed to cover 95% of the breast planning target volume with the 95% isodose and 80% of the regional nodal planning target volume with the 95% isodose. Treatment was delivered using a simple field-in-field intensity-modulated radiotherapy technique applying multiple beam segments to optimise dose homogeneity. The dose schedule was either 40 Gy in 15 fractions (over 3 weeks) or 42·72 Gy in 16 fractions (over 3·2 weeks). In keeping with International Commission on Radiation Units criteria,^22^ hotspots of greater than 107% were limited to less than 2 cm^3^. Real-time electronic portal imaging was performed on fractions one to three and then weekly thereafter. Skin radiation toxicity assessment was done preoperatively using the Radiation Therapy Oncology Group toxicity grading system.^23^

At surgery, breast and nodal tissue samples were collected for histology and stored in the Royal Marsden Biobank to be used for future translational studies. Surgical complications were recorded at 24 h, then at 4, 8, and 12 weeks postoperatively, and were managed according to local unit protocols. Wound status was recorded as either open (>1 cm width requiring a dressing) or closed and, if open, was followed up until healed. Mastectomy skin flap necrosis was defined as black, non-viable, or dead skin, and fat necrosis within the flap as a palpable lump with or without sonographic or histological evidence. Aesthetic assessments were done at baseline (preoperatively), and at 3 and 12 months after surgery for patients at the Royal Marsden site only. Aesthetic assessments involved completion of a BREAST-Q questionnaire (a validated outcome measure of patient satisfaction)^24^ and three-dimensional surface imaging (3D-SI), using the VECTRA XT capture system (Canfield Scientific, Parsippany, NJ, USA). 3D-SI was subjected to panel evaluation using a scale developed for breast reconstruction through Delphi consensus.^25^

### Outcomes

The primary endpoint was the proportion of open breast wounds of greater than 1 cm width at 4 weeks after surgery that required dressing or packing.

Prespecified secondary outcomes included the presence of an open breast wound greater than 1 cm width at 8 and 12 weeks after surgery; DIEP flap failure rate (defined as partial or whole flap necrosis necessitating flap debridement or removal); patient-reported satisfaction with the breast reconstruction (BREAST-Q reconstructive module) before surgery, and 3 months and 12 months after surgery; and panel assessment of 3D-SI. Differences in surface and volume symmetry between the reconstructed and non-reconstructed breast, another secondary endpoint, were measured using 3D-SI and these data will be published separately. A commercial applanation tonometry system could not be sourced, hence differences in compressibility between the reconstructed and non-reconstructed breast (secondary endpoint) will not be reported. A final secondary endpoint, associations between open wounds and preoperative variables, was not computed given the low event rate.

### Statistical analysis

The study was not powered for a cancer outcome and the planned sample size was initially set at 20 patients to allow the PRADA Trial Management Group to ascertain the technical feasibility of schedule reversal with a view to subsequent cohort expansion, to perform the current feasibility study, if treatment sequencing was deemed to be feasible. Once it was clear that schedule delivery was feasible clinically, we expanded our sample to more than 30 patients based on guidance from the National Institute for Health Research (NIHR) on the design of feasibility studies. Using the simple asymptotic method for 30 patients, we used a two-sided 95% CI for a single proportion of patients with open wounds at 4 weeks after surgery. A proportion of less than 5% was assumed to be observed within a 95% CI of plus or minus 7·8% or within range from 0–12·8%. Therefore, based on these boundaries, the true open wound rate is unlikely to be more than 12·8%. Descriptive statistics were used for demographic data and clinical outcomes. Continuous variables are presented as median (IQR). Categorical variables are reported as the absolute number of patients and relevant proportions. 95% CIs were computed using the binomial exact calculation.

BREAST-Q questionnaires were analysed using the Q-Score software (version 2.0). The resulting Q-score ranges from 0 to 100, with 100 being the best possible score. 3D-SI global panel scores were assessed on a scale from 1 (very poor) to 5 (excellent). Treatment pathway durations were computed for all patients. There were no prespecified subgroup analyses.

Post-hoc exploratory analyses were the proportion of patients who had unplanned return to theatre and mastectomy skin flap necrosis, treatment pathway durations, and the following oncological outcomes: pathological complete response, local recurrence, regional nodal recurrence, disease-free survival, and overall survival, which were analysed with descriptive statistics. Pathological complete response was defined as the absence of invasive or in-situ cancer in breast and axillary nodes (ypT0ypN0) and was recorded separately for the breast and axilla as a binary outcome (yes or no). Local recurrence was defined as disease recurrence in the chest wall. Regional nodal recurrence was defined as recurrence in regional lymph nodes, including the axilla, supraclavicular fossa, or internal mammary nodes. Disease-free survival was defined as the interval between the date of diagnosis and the first breast cancer recurrence, with the event being any breast cancer recurrence (locoregional or systemic) or death. Overall survival was defined as the interval from the date of diagnosis until death from any cause. Disease-free and overall survival analyses were conducted using the Kaplan-Meier method and the binomial exact method was used to compute 95% CI.

Statistical data analysis was conducted at Imperial College London, London, UK, using IBM SPSS Statistics (version 27). This study is registered with ClinicalTrials.gov, NCT02771938, and is closed to recruitment.

### Role of the funding source

The funders of the study had no role in study design, data collection, data analysis, data interpretation, or writing of the report.

## Results

Between Jan 25, 2016, and Dec 11, 2017, 33 patients were enrolled (19 at the Royal Marsden NHS Foundation Trust and 14 at the Imperial College Healthcare NHS Trust). Baseline demographics and clinicopathological data are reported in table 1. Preoperative radiotherapy was delivered before primary mastectomy in 31 (94%) of 33 patients or before completion of mastectomy in two (6%) patients. Justifications for mastectomy and preoperative radiotherapy, and data regarding the technical aspects of oncological and reconstructive surgery are reported in the appendix (pp 5–8). All patients were assessed for the primary outcome, and there were no dropouts.

**Table 1:** Baseline characteristics

		**Patients (n=33)**
Age, years		48·0 (13·0)
Body-mass index, kg/m^2^		28·0 (5·3)
Ethnicity		
	White	29 (88%)
	Afro-Caribbean	1 (3%)
	Asian	1 (3%)
	Arabic	2 (6%)
Comorbidity		
	Diabetes	1 (3%)
	Smoking	3 (9%)
Tumour laterality		
	Left	13 (39%)
	Right	20 (61%)
Tumour subtype		
	Invasive ductal carcinoma	27 (82%)
	Invasive lobular carcinoma	6 (18%)
Tumour stage		
	T2	11 (33%)
	T3	17 (52%)
	T4b (involved nipple areolar complex)	5 (15%)
Receptor status		
	Oestrogen receptor-positive	25 (76%)
	Progesterone receptor-positive	22 (67%)
	HER2-positive	8 (24%)
	Triple-negative breast cancer	4 (12%)
Nodal stage		
	0	8 (24%)
	I	21 (64%)
	II	3 (9%)
	III	1 (3%)

Data are median (IQR) or n (%).

An open wound greater than 1 cm width requiring a dressing at 4 weeks after surgery was noted in four (12·1%, 95% CI 3·4–28·2) of 33 patients (table 2); the wounds were minor and managed conservatively with dressings and antibiotics in three (9%) patients, and one (3%) patient required reoperative intervention for debridement and skin graft. The only other unplanned return to theatre was within 24 h, to assess a congested flap that required no intervention.

**Table 2:** Postoperative complications by time since surgery (n=33)

	**<24 h**	**4 weeks**	**8 weeks**	**12 weeks**	**Total**
Open wound >1 cm width requiring a dressing (primary outcome)	0	4 (12%)	4 (12%)	0	4 (12%)
Unplanned return to theatre	1 (3%)	1 (3%)	0	0	2 (6%)
Mastectomy skin envelope necrosis	0	4 (12%)	0	0	4 (12%)
DIEP flap failure	0	0	0	0	0
Fat necrosis	0	1 (3%)	5 (15%)	0	6 (18%)

Data are n (%). DIEP=deep inferior epigastric perforator.

With regard to secondary outcomes, four (12%) of 33 patients at 8 weeks after surgery and none at 12 weeks had an open breast wound greater than 1 cm requiring a dressing. After a median follow-up of 23·6 months (IQR 8·2), there were no DIEP flap failures. 17 (89%) of 19 patients at the Royal Marsden NHS Foundation Trust participated in aesthetic assessments. BREAST-Q questionnaires were completed by 14 (82%) of 17 patients preoperatively, 13 (76%) at 3 months after surgery, and 12 (71%) at 12 months after surgery. 3D-SI images for panel evaluation were obtained for 13 patients at 3 months and 12 at 12 months after surgery. The median satisfaction with BREASTS Q-score was 48·0 (IQR 5·0) at baseline, 73·0 (14·0) at 3 months after surgery, and 77·0 (15·0) at 12 months after surgery (table 3). The median 3D-SI global panel score was 3·9 (IQR 0·6) 5·0 at 3 months and 4·3 (0·7) at 12 months after surgery.

**Table 3:** BREAST-Q scores

	**Patients who completed questionnaire (n=17)**	**Satisfaction with breasts**	**Satisfaction with outcome**	**Psychosocial wellbeing**	**Physical wellbeing (chest)**	**Physical wellbeing (abdomen)**	**Sexual wellbeing**
Baseline (preoperative)	14 (82%)	48·0 (5·0)	NA	60·0 (26·0)	77·0 (21·5)	91·5 (17·0)	47·5 (20·3)
3 months after surgery	13 (76%)	73·0 (14·0)	100·0 (25·0)	79·0 (15·0)	63·0 (19·8)	70·0 (17·0)	54·0 (18·0)
12 months after surgery	12 (71%)	77·0 (15·0)	100·0 (16·8)	76·0 (29·8)	83·0 (13·3)	79·0 (30·0)	57·0 (50·8)

Data are n (%) or median (IQR). NA=not applicable.

Treatment timelines are summarised in table 4. Three (9%) of 33 patients did not receive neoadjuvant chemotherapy and were excluded from computations of neoadjuvant chemotherapy to preoperative radiotherapy and neoadjuvant chemotherapy to mastectomy timelines. Overall, 27 (82%) of 33 patients had surgery within 4 weeks of preoperative radiotherapy, and all had surgery within 6 weeks.

**Table 4:** Treatment timelines

	**Median (IQR), days**	**Patients assessed, n**
Time from completion of neoadjuvant chemotherapy to preoperative radiotherapy	30·0 (30·0)	30
Time from preoperative radiotherapy to mastectomy	20·0 (11·0)	33
Time from completion of neoadjuvant chemotherapy to mastectomy	69·0 (32·0)	30
Time from diagnosis to mastectomy	230·0 (76·0)	33

Treatment dates and timelines were available for all patients; since three patients did not receive neoadjuvant chemotherapy, these patients have been excluded from computations of time from completion of neoadjuvant chemotherapy to preoperative radiotherapy and time from completion of neoadjuvant chemotherapy to mastectomy.

Breast pathological complete response was reported in seven (21%, 95% CI 8·98–38·91) of 33 patients and axillary pathological complete response in six (26%, 10·23–48·41) of 23 patients. Pathological complete response rates varied according to immunophenotype, with greater rates in triple-negative and HER2-positive cancers than in luminal-type breast cancers (appendix p 9). Management of the axilla and final pathological nodal stage is reported in the appendix (pp 10–11).

**Figure 2:**
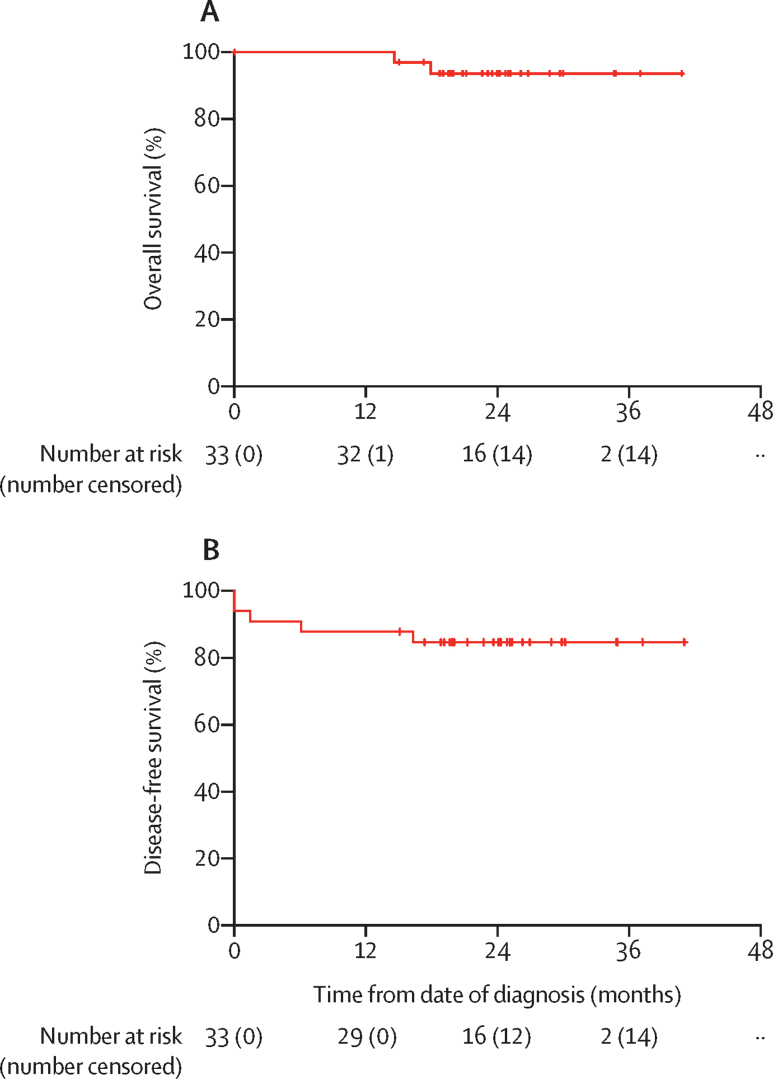
Kaplan-Meier survival curves (A) Overall survival. (B) Disease-free survival. Ticks indicate censored patients.

After a median follow-up of 23·6 months (IQR 8·2), there were no local recurrences, no regional nodal recurrences, four (12%) of 33 patients had distant metastatic disease, and two (6%) died from breast cancer. Overall survival (two deaths in 33 patients) was 93·9% (95% CI 79·7–99·2; figure 2A) and disease-free survival (five events in 33 patients) was 84·8% (68·1–94·9; figure 2B).

Most patients (30 [91%] of 33) received neoadjuvant chemotherapy as per institutional guidelines, of whom ten (33%) required a dose reduction (table 5). With regard to preoperative radiotherapy, 29 (88%) of 33 patients received 40 Gy in 15 fractions and four (12%) received 42·72 Gy Gy in 16 fractions. Regional nodal radiotherapy was delivered to the axilla in 12 (36%) of 33 patients, supraclavicular fossa in 29 (88%), and internal mammary nodes in 11 (33%; table 5). Four (12%) of 33 patients received no regional nodal radiotherapy. With regard to radiotherapy skin toxicity, one (3%) of 33 patients had grade 0, 22 (67%) had grade 1, and nine (27%) had grade 2 adverse events (appendix p 12). One (3%) patient experienced confluent moist desquamation (grade 3 toxicity). There were no reports of grade 4 toxicity and no patients discontinued due to treatment-related toxicity. There were no serious adverse events and no treatment-related deaths.

**Table 5:** Neoadjuvant chemotherapy and preoperative radiotherapy treatment details

		**Patients (n=33)**
Neoadjuvant chemotherapy		30 (91%)
Chemotherapy dose reduction		
	Yes	10 (33%)*
	No	19 (63%)*
	Not known	1 (3%)*
Preoperative radiotherapy to chest wall		33 (100%)
Radiotherapy fractionation schedule		
	40 Gy in 15 fractions	29 (88%)
	42·72 Gy in 16 fractions	4 (12%)
Regional nodal irradiation		
	Level I and II (axilla)	12 (36%)
	Level III and IV (supraclavicular fossa)	29 (88%)
	Internal mammary nodes	11 (33%)
	None	4 (12%)

Data are n (%).

* n=30.

## Discussion

In this multicentre, prospective study, we showed that preoperative radiotherapy followed by skin-sparing mastectomy and immediate microvascular DIEP flap reconstruction is technically feasible and safe. Crucially, the rate of open wounds, mastectomy skin necrosis, fat necrosis, and unplanned returns to the operating theatre were low, with no DIEP flap failures. These results align with UK national reported outcomes following autologous breast reconstruction,^8^ attesting to substantial expertise in microsurgery in the UK. 12 months after surgery, the patients in this study reported high satisfaction with the breast reconstruction, and very good aesthetic outcomes were observed on panel assessment.

The primary endpoint, an open breast wound at 4 weeks after surgery, was chosen as a simple but objective, early, and easily measurable safety outcome. Given the longstanding concerns regarding wound healing following radiotherapy,^26^ open wound rate is an important safety outcome. Open wounds delay adjuvant therapy,^5^ might require secondary surgical intervention,^27^ prolong treatment, and affects the breast aesthetic. This outcome is highly relevant to multidisciplinary teams whose preferred reconstructive strategy is expander or implant-based, because the safety of preoperative radiotherapy with respect to postoperative wound complications is even more crucial in this scenario. Wound measurement sought to minimise observer bias in the reporting of open wounds. Few open breast wounds were observed, and all were preceded by mastectomy skin necrosis. These patients had concurrent axillary dissection; three of these procedures were done through circumareolar incisions. The patient who required debridement and a skin graft was an active smoker with a body-mass index greater than 30 kg/m^2^ and received a level 3 dissection through the areolar incision. The combination of comorbidities and traction on the skin flap to enable access to the axilla might have contributed to skin necrosis. A separate axillary incision could be considered for individuals at high risk of skin necrosis. The interplay between radiation-related vascular injury and microvascular disease caused by smoking might increase the risk of skin necrosis.^28^ Nevertheless, the skin necrosis rate in this study was in keeping with other preoperative radiotherapy studies (3·0–12·5%),^16^, ^17^, ^18^, ^29^ and similar to a large prospective cohort study that reported a skin necrosis rate of 14%.^30^

Historical concerns regarding preoperative radiotherapy include the perception of increased postoperative surgical wound complications, but several contemporary studies refute this idea.^15^, ^17^, ^29^, ^31^ The largest of these, a review of the American College of Surgeons National Surgical Quality Improvement Program database, assessed the impact of preoperative radiotherapy on 30-day postoperative morbidity after mastectomy with or without breast reconstruction.^31^ In both the mastectomy-only and immediate reconstruction groups, preoperative radiotherapy was not associated with an increased risk of complications after multivariate regression analysis (9·4% with preoperative radiotherapy *vs* 11·1% without preoperative radiotherapy for mastectomy only; p=0·48; and 14·7% *vs* 11·2% for immediate reconstruction; p=0·22).^31^ Giacalone and colleagues^16^ reported no difference in rates of skin necrosis in 648 patients who had mastectomy and implant-assisted latissimus dorsi flap reconstruction with or without preoperative radiotherapy.^16^ Similarly, both Zinzindohoué and colleagues^18^ and Paillocher and colleagues^29^ observed low rates of skin necrosis (6% and 5·3%, respectively) in patients receiving neoadjuvant chemoradiotherapy followed by mastectomy and implant-assisted latissimus dorsi flap reconstruction. Although Monrigal and colleagues^17^ observed higher rates of skin flap necrosis (within 30 days) in a transverse rectus abdominis musculocutaneous subgroup, the overall skin necrosis rate was 7·6%.

The study most similar and contemporaneous to PRADA also showed low rates of postoperative complications in patients receiving preoperative radiotherapy followed by skin-sparing or partial skin-sparing mastectomy.^15^ In this study, Grinsell and colleagues^15^ adopted a delayed-inset approach in 15 (52%) of 29 patients 3 days after mastectomy (all with the same surgeon). In these patients, the DIEP skin paddle was preserved deep to the breast skin envelope for use as a lifeboat. Importantly, seven (47%) of 15 patients required conservative debridement of the skin edge at inset.^15^ By contrast, in our cohort, all patients (with one exception) had skin-sparing mastectomy. In PRADA, surgery was performed by 11 breast surgeons and seven plastic surgeons across two institutions with different axillary management and radiotherapy protocols, highlighting the reproducibility and replicability of our findings.

Importantly, patients reported high satisfaction with the breast reconstruction 12 months after surgery and aesthetic outcomes were favourable upon panel assessment. Although the literature on aesthetic evaluation has many limitations with heterogenous populations and methods, the satisfaction with breasts Q-score for the PRADA population was higher than in a study of post-mastectomy radiotherapy, in which the median Q-score for irradiated autologous reconstructions was 64 (IQR 20) after a median follow-up of 27·5 months (IQR 22·8–42·2).^32^

Patients received surgery within approximately 1 month from radiotherapy delivery. The ideal time from preoperative radiotherapy to surgery is not well defined and varies widely in the literature.^29^, ^33^ The period of 4–6 weeks is calculated with the intent of allowing resolution of acute inflammation, as well as allowing for tumour regression, while minimising the early fibrotic changes in the surgical field. Surgery performed more than 6 weeks after radiotherapy increases microvascular complication rates,^20^ which are related to venous thrombotic complications in recipient veins.

Neoadjuvant chemotherapy followed by preoperative radiotherapy was conceived to improve rates of tumour response, with the rationale that tumour cells surviving after chemotherapy would be sterilised by irradiation due to synergistic effects. In the present study, the relatively low rates of pathological complete response reflect the preponderance of oestrogen receptor-positive HER2-negative breast cancers and, as expected, higher response rates were observed in the basal-type and HER2-positive cancers. The PRADA cohort had high-risk disease (stage T3 or T4 and lymph node positive), so it is encouraging that no local recurrence or regional nodal recurrence events were observed. Interestingly, other preoperative radiotherapy studies also report low rates of local recurrence (0·9%^29^ and 2·4%^17^).

Oncological outcome findings in this study were similar to those observed in other preoperative radiotherapy series,^15^ and in case-control studies comparing survival between patients who received preoperative radiotherapy and post-mastectomy radiotherapy.^14^, ^33^ For example, Brackstone and colleagues^14^ showed no significant difference in disease-free survival (preoperative radiotherapy 81% *vs* post-mastectomy radiotherapy 69%; p=0·19) or overall survival (89% *vs* 74%; p=0·16) between patients receiving chemoradiotherapy and matched controls. Gerlach and colleagues^33^ reported improved overall survival in patients who received preoperative radiotherapy (median 19 months) compared with those who received post-mastectomy radiotherapy (13 months).

This study has several limitations. A powered endpoint was not provided per se; however, the outcomes are important for sample size estimates in a randomised trial. Non-probabilistic sampling might have led to selection bias. Although the intention was for patients to receive neoadjuvant chemotherapy, not all patients received chemotherapy per protocol, but preoperative radiotherapy might have a role in circumstances where preoperative systematic therapy might not be required (eg, locally advanced oestrogen receptor-positive disease). Pragmatism in radiotherapy delivery, such as decisions regarding the use of bolus, would require greater uniformity in a randomised trial, especially given recent Delphi consensus suggesting greater selectivity in the use of bolus following mastectomy and breast reconstruction.^34^ However, heterogeneity in radiotherapy did not affect outcomes, because complications were not restricted to one site, radiation oncologist, or surgeon. Moreover, because radiotherapy schedules are known to vary between centres and internationally, heterogeneity in radiotherapy enhances external validity. There is limited information on the adverse effects of radiotherapy on the preserved breast envelope, except for reports of mastectomy skin flap necrosis. Future trials will evaluate the potential long-term sequelae of preoperative radiotherapy, including fibrosis and contracture of the skin envelope, pigmentation, erythema, and alterations in breast sensation.

Preoperative radiotherapy followed by skin-sparing mastectomy and DIEP flap reconstruction is technically feasible, with low rates of surgical complications and good short-term oncological outcomes. Further evaluation in a randomised trial of preoperative radiotherapy versus conventional post-mastectomy radiotherapy in breast reconstruction is now required to compare oncological and quality-of-life outcomes.

## Data sharing

The approval from the ethics committee does not include permission to make the underlying data materials available.

## Declaration of interests

PTRT reports personal fees from Stryker Surgical and Cytoveris. DRL is an advisory board member for CMR Surgical. JEH reports personal fees from Stryker Surgical. All other authors declare no competing interests.
