# Supplementary material for: Primary radiotherapy and deep inferior epigastric perforator flap reconstruction for patients with breast cancer (PRADA): a multicentre, prospective, non-randomised, feasibility study
Source: Lancet Oncol. 2022 May;23(5):682–90. doi: 10.1016/S1470-2045(22)00145-0 (PMC9630150; doi:10.1016/S1470-2045(22)00145-0)
Supplement: Supplementary appendix [file mmc1.pdf]

THE LANCET
Oncology

## Supplementary appendix

This appendix formed part of the original submission and has been peer reviewed. We post it as supplied by the authors.

Supplement to: Thiruchelvam PTR, Leff DR, Godden AR, et al. Primary radiotherapy and deep inferior epigastric perforator flap reconstruction for patients with breast cancer (PRADA): a multicentre, prospective, non-randomised, feasibility study. *Lancet Oncol* 2022; published online April 7. https://doi.org/10.1016/S1470-2045(22)00145-0.

## Supplementary Materials (SM)

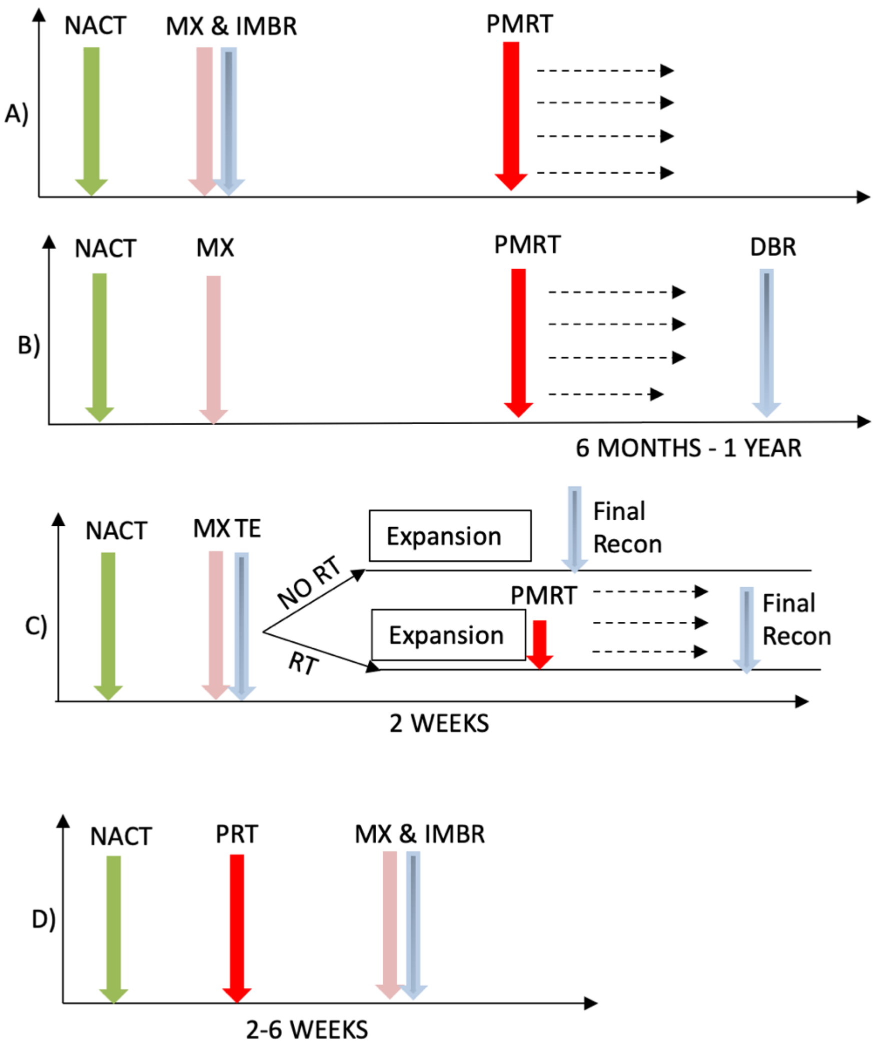


**SM 1. Comparative pathways for patients with LABC requiring neoadjuvant chemotherapy (NACT) and preoperative radiotherapy PRT, and desiring breast reconstruction.** Treatment pathway options include: a) Standard pathway - NACT followed by mastectomy (MX) and immediate reconstruction and then post-mastectomy radiotherapy (PMRT) with potential for delay (arrows); b) Delayed pathway - NACT followed by simple mastectomy and then PMRT and delayed reconstruction; c) the "Delayed Immediate" pathway with temporary tissue expander to be converted to autologous reconstruction down-stream and finally d) the proposed PRADA pathway with preoperative radiotherapy (PRT) followed by mastectomy and immediate reconstruction (2-6 weeks).

| SM 2. Indications for PRADA Preoperative Radiotherapy |
| --- |
| **Chest wall.** All patients recruited received preoperative chest wall radiotherapy |
| • cN2-3c<br>• cN≥ 4 lymph nodes<br>• cN1 + other high risk features (age, tumour biology, grade, size etc)<br>• cT3N0 |
| **Supraclavicular fossa (SCF*) (Levels III-IV )** |
| • N3c<br>• cN2-3b<br>• cN≥ 4 lymph nodes<br>• Axillary lymph node measuring ≥2cm<br>• Persistent axillary disease after neoadjuvant chemotherapy (patients with persistent ER positive HER2 negative disease with low volume axillary disease persisting after chemotherapy who undergo a level II or III dissection may benefit little)<br>• Fields matched to most superior and medial axillary marker clip if axillary clearance performed prior chemotherapy/radiotherapy |
| **Internal mammary** |
| • cN3b<br>• cN2-3a/c<br>• cN≥ 4 lymph nodes<br>• Medial tumours with adverse biology |
| **Axilla and SCF*(Levels I-IV)** |
| • Radiotherapy was not recommended after axillary dissection/clearance except when the surgeon did not achieve macroscopic disease clearance.<br>• Radiotherapy was permitted after a positive SLNB/targeted axillary dissection instead of axillary node clearance (the preferred treatment). |
| c=Clinical. N=Node. * The term 'SCF' is used to denote the anatomical area above the level of axillary dissection/clearance. This typically means axillary levels III and IV. Indications adapted from guidance on postmastectomy radiotherapy. (1) |

| SM3. Patient Characteristics, Demographic and Clinicopathological Data | |
|---|---|
| **Demographic variables** | **Data [median (IQR)]** |
| Age [years] | 48·0 (13·0) |
| Body Mass Index [kg/m2] | 28·0 (5·3) |
| | |
| **Ethnicity** | **Data n (%) of N** |
| Caucasian | 29 (87·9) of 33 |
| Afro-Caribbean | 1 (3·0) of 33 |
| Asian | 1 (3·0) of 33 |
| Arabic | 2 (6·0) of 33 |
| **Comorbidity** | |
| Diabetes mellitus n(%) | 1 (3·0) of 33 |
| Smoking n(%) | 3 (9·1) of 33 |
| **Tumour-related variables** | **Data n (%) of N** |
| **Tumour laterality** | |
| Left n(%) | 13 (39·4) of 33 |
| Right n(%) | 20 (60·6) of 33 |
| **Tumour Subtype** | |
| IDC | 27 (81·8) of 33 |

| | |
|---|---|
| ILC | 6 (18·2) of 33 |
| **Tumour Stage** | |
| T2 | 11 (33·3) of 33 |
| T3 | 17 (51·5) of 33 |
| T4b (involved NAC) | 5 (15·2) of 33 |
| **Receptor Status (% Positive)** | |
| ER | 25 (75·8) of 33 |
| PR | 22 (66·7) of 33 |
| HER2 | 8 (24·2) of 33 |
| TNBC | 4 (12·1) of 33 |
| **Nodal Stage** | |
| 0 | 8 (24·2) of 33 |
| I | 21 (63·6) of 33 |
| II | 3 (9·1) of 33 |
| III | 1 (3·0) of 33 |

IDC – invasive ductal carcinoma, ILC – invasive lobular =carcinoma, NAC=nipple areolar complex.

## SM 4 Justification for Mastectomy and Radiotherapy

In 31 (93·9%) of 33 patients, skin sparing mastectomy was the primary surgical procedure and in 2 (6·1%) completion mastectomy was indicated after positive margins following an attempt at breast conserving surgery. Justifications for mastectomy are summarised for each patient in supplementary material 4. Regarding the two patients who received completion mastectomy after failed breast conserving surgery, one was found to have a 58mm invasive lobular cancer with involved margins and 3/33 positive nodes after initial surgery, and the second had multifocal invasive disease (a 24mm focus and a separate 23mm focus with high grade ductal carcinoma in situ at the resection margins and 6/15 positive nodes). In these cases, re-excision of margins was deemed technically infeasible, and radiotherapy was indicated for either tumour size and/or nodal involvement (see Table SM4).

**SM4. Indication for mastectomy and justification for preoperative radiotherapy for each PRADA case**

| Case Number | Indication for mastectomy | Justification for PRT |
|---|---|---|
| 1 | Multifocal | N2 |
| 2 | T3 | T3 |
| 3 | T3 | T3 |
| 4 | T3 | T3 |
| 5 | Multifocal | T3 |
| 6 | Multifocal | N1 |
| 7 | Tumour/breast ratio | N1 |
| 8 | T3 | T3 |
| 9 | Tumour/breast ratio | N1 |
| 10 | T3 + failed BCS | T3 |
| 11 | T4 | T4 |
| 12 | T4 | T4 |
| 13 | Tumour/breast ratio | N2 |
| 14 | Multifocal | N1 |
| 15 | Tumour/breast ratio | N1 |
| 16 | T3 | T3 |
| 17 | T4 | T4 |
| 18 | T4 | T4 |
| 19 | T4 | T4 |
| 20 | Multifocal | T3 |
| 21 | T3 | T3 |
| 22 | Multifocal | T3 |
| 23 | Multifocal | N1 on SNB |
| 24 | T3 | T3 |
| 25 | Multifocal + failed BCS | T3 on WLE |

| | | |
|---|---|---|
| 26 | T3 | T3 |
| 27 | T3 | T3 |
| 28 | Multifocal | T3 |
| 29 | T3 | T3 |
| 30 | Multifocal | T3 |
| 31 | T3 | T3 |
| 32 | Multifocal | N3 |
| 33 | Tumour/breast ratio | N2 |

PRT = preoperative radiotherapy, WLE=wide local excision, BCS=breast conserving surgery, SNB=sentinel node biopsy, T=tumour stage, N=nodal stage.

**SM 5. Summary of oncologic and reconstructive surgical treatment**

| **Oncological surgery** | **Data n (%) of N or Median (IQR)** |
|---|---|
| Primary mastectomy (%) | 31 (93·9) of 33 |
| Mastectomy for positive margins (%) | 2 (6·1) of 33 |
| Breast weight [median(IQR) gm] | 574 (490) |
| **Axillary surgery** | |
| SLNB (%) | 13 (39·4) of 33 |
| Positive SLNB (%) | 6 (46·2) of 13 |
| ALND (%) | 23 (69·7) of 33 |
| Separate incision ALND (%) | 15 (65·2) of 23 |
| **Reconstructive Surgery** | |
| Immediate flow (%) | 30 (90·9) of 33 |
| Need for anastomosis revision (%) | 3 (9·1) of 33 |
| Vessel size / venous coupler [median(IQR) mm] | 2·5 (1) |
| Ischaemic time [median(IQR) min] | 39·5 (22) |
| Flap weight [median(IQR) gm] | 653 (402) |
| **Peri-operative outcomes** | |
| Total operative time [median(IQR) min] | 480 (120) |
| Blood loss [median(IQR) ml] | 375 (300) |
| Length of stay (days) | |
| 3 to 4 (%) | 13 (39·4) of 33 |

| | |
|---|---|
| 5 to 6 (%) | 15 (45·4) of 33 |
| 7 to 8 (%) | 5 (15·1) of 33 |

ALND – axillary lymph node dissection, SLNB – sentinel lymph node biopsy= data are proportion receiving SLNB. Positive SLNB=data are proportion of SLNB patients with positive sentinel lymph nodes.

**SM 6. Breast and axillary pathological complete response rate by molecular subtype**

| Molecular subtype | Breast pCR n (%) of N | Axilla pCR n (%) of N |
|---|---|---|
| Luminal-type | 3 (12·5) of 24 | 2 (12·5) of 16 |
| HER2-enriched | 2 (40·0) of 5 | 2 (50·0) of 4 |
| TNBC | 2 (50·0) of 4 | 2 (66·7) of 3 |
| All | 7 (21·2) of 33 | 6 (26·1) of 23 |

pCR - pathological complete response, TNBC - triple negative breast cancer, HER2 - human epidermal growth factor receptor.

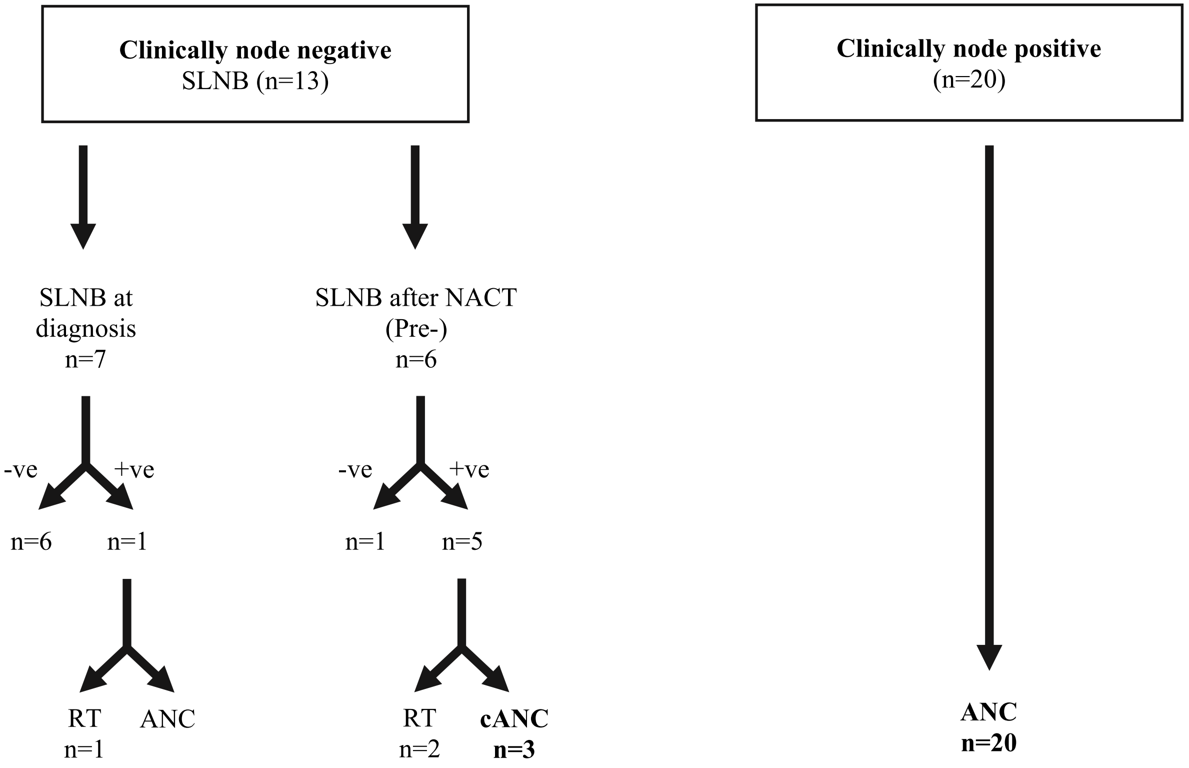


**SM 7. Axillary management of patients in PRADA trial.** Patients with clinically node negative disease received sentinel lymph node biopsy (SLNB) at diagnosis or after neoadjuvant chemotherapy (NACT) but before preoperative radiotherapy (Pre-). Patients with clinically node positive disease (n=20) underwent "upfront" axillary nodal dissection (ANC). Three patients with residual disease on SLNB after NACT received completion axillary dissection (cANC) (n=3).

**SM 8. Final pathological nodal stage**

| Pathological outcome ALND (excluding +SNB) - n (%) of N | pN0 | pN1 (1-3) | pN2 (≥4-10) | pN3 (≥10) |
|---|---|---|---|---|
| | 6 (26·1) of 23 | 8 (34·8) of 23 | 5 (21·7) of 23 | 4 (17·4) of 23 |

ALND – Axillary Nodal Dissection, SNB – Sentinel Node Biopsy. Data was available for all 23 patients receiving ALND.

| SM9. Radiotherapy skin toxicity adverse effect profile | | |
|---|---|---|
| RTOG Grade | RTOG Descriptor | n (%) of N |
| 0 | No change over baseline | 1 (3·0) of 33 |
| 1 | Follicular, faint, or dull erythema | 18 (54·5) of 33 |
| | Dry desquamation | 4 (12·1) of 33 |
| 2 | Tender or bright erythema | 5 (15·1) of 33 |
| | Patchy moist desquamation | 4 (12·1) of 33 |
| 3 | Confluent moist desquamation other than skin folds | 1 (3·0) of 33 |
| 4 | Ulceration, Haemorrhage or Necrosis | 0 (0) of 33 |
| RTOG - Radiation Therapy Oncology Group | | |

**Summary of Sites, Site PIs, and Trial Recruitment**

Site: Royal Marsden NHS Foundation Trust
Site PI: Fiona MacNeill
Number of patients recruited: 19

Site; Imperial College Healthcare NHS Trust
Site PI: Dimitri J Hadjiminas
Number of patients recruited: 14

**Appendix References**


1. Recht A, Edge SB, Solin LJ, Robinson DS, Estabrook A, Fine RE, et al. Postmastectomy radiotherapy: clinical practice guidelines of the American Society of Clinical Oncology. J Clin Oncol. 2001;19(5):1539-69.

**Primary Radiotherapy And DIEP flAp reconstruction**

**The PRADA Study**

| | |
|---|---|
| **Protocol Reference:** | CCR 4328 |
| **Version Number & Date:** | 2.2 ( with REC revisions) |
| **Effective Date:** | 19/8/2015 |
| **Superseded Version Number & Date (If applicable)** | 2.1 |

## Chief Investigator: Miss Fiona MacNeill

**Contents Page**


1. Lay summary
2. Background
3. Study Aims & Objectives
4. Study design
5. Endpoints
6. Inclusion/Exclusion Criteria
7. Methodology
8. Data acquisition
9. Data analysis
10. Study organisation
11. Evaluation
12. Adverse events
13. Statistics
14. Patient and Public Involvement (PPI)
15. Regulatory & Ethics Committee Approval
16. Data Handling and Record Keeping
17. Financing, Indemnity & Insurance
18. Publication Policy
19. Abbreviations
20. References
21. Appendices
    - A - Patient information sheet (PIS)_RMH_V2
    - B - Consent form_RMH_V2
    - C - CRF PRADA Study_v1
    - D –Qualitative (3D Photography/Applanation) CRF_RMH_v1
    - E - BREAST-Q CRF_RMH_v1
    - F - BREAST Q-ReconstructiveModule-Questionnaire

## 1. Lay Summary

Many women with breast cancer now live for decades after their breast cancer treatment. In view of this, modern breast reconstruction surgery after mastectomy for breast cancer aims to reproduce as natural a breast shape as possible. Keeping a natural breast appearance has been shown to be very important to a woman's emotional and psychological recovery.

Breast cancer treatment often includes a combination of surgery, chemotherapy, radiotherapy, anticancer tablets such as Tamoxifen, and newer targeted drugs such as Herceptin. Radiotherapy is usually given after surgery. However, radiotherapy after mastectomy and breast reconstruction can damage the 'new' breast giving a less good breast shape and appearance in the longer term. Also, if recovery is slow following surgery, the radiotherapy is delayed which may reduce its effectiveness. Changing the order of treatments has been shown to be safe and effective for chemotherapy, Herceptin and anticancer tablets but we have very little information on giving radiotherapy before breast cancer surgery.

We want to find out if giving radiotherapy before mastectomy and reconstruction alters surgical complication rates. We are also evaluating the appearance of the reconstructed breast when radiotherapy is given before surgery.

### 2.1 Background

Cancer outcomes are equivalent regardless of the order of systemic treatments and surgery (1) with a trend towards improved overall survival in women age <50 years receiving chemotherapy before surgery. Adjuvant post-mastectomy chest wall RT has been shown to have both a local and survival benefit particularly in high risk patients (2). Accordingly, patients with T3/T4 breast cancer and/or with a significant burden of axillary disease commonly now receive a treatment sequence comprising primary chemotherapy followed by mastectomy and immediate autologous reconstruction, increasingly using abdominal fat (DIEP reconstruction), and finally adjuvant radiotherapy to the affected chest wall +/- supraclavicular fossa.

There are precedents for the use of upfront (neoadjuvant) radiotherapy (NART) followed by complex cancer surgery. For example, in rectal cancer, there is substantial evidence for the use of neoadjuvant chemotherapy and radiotherapy followed by aggressive surgical excision as the standard of care in patients with a threatened or involved circumferential margin. Short-course preoperative radiotherapy has been tested in multiple trials in rectal cancer, including the Swedish Rectal Cancer Trial, Dutch Colorectal Cancer Group Study and more recently the Medical Research Council CR07 trial (3). All three studies demonstrated better local control and improved disease-free and overall survival. Flap reconstruction of the perineum at the time of abdomino-perineal resection is well described as a method to reduce perineal morbidity and is indicated when primary closure cannot be achieved after wide local resection. By transferring a bulk of vascularized soft tissue into the irradiated pelvis, flap reconstruction has been shown to reduce infection rates, fill pelvic dead space, prevent wound dehiscence, and reduce time to healing. 'Short course' pre-operative rectal radiotherapy: surgery is generally undertaken 7-10 days after completion of radiotherapy with an acceptable impact on post-operative complication rate (3).

There is one published series of NART in breast cancer reporting an acceptable post-operative complication rate (4). Following on from this, surgeons and clinical oncologists from Imperial College and the Royal Marsden have begun to develop a limited experience of mastectomy and DIEP reconstruction 14 days following completion of radiotherapy (10 cases, no significant post-operative complications). This non-randomised phase I study sets out to formally evaluate the safety of reversing the order of mastectomy plus immediate DIEP flap reconstruction and adjuvant radiotherapy, with a view to a subsequent randomised controlled trial testing local control and cosmetic outcomes.

**3.1 Aim**
The aim of this study is to determine the surgical outcomes of radiotherapy prior to surgery.

**3.2 Objectives**
Radiotherapy (RT) prior to mastectomy with immediate DIEP flap reconstruction:

- Is feasible with equivalent acute complications rates to standard mastectomy and immediate DIEP flap reconstruction performed prior to RT
- Will improve the long-term aesthetic outcome of mastectomy and immediate DIEP flap reconstruction in patients requiring radiotherapy
- Avoids delays to radiotherapy after surgery because of wound healing issues.
- Will ultimately increase immediate reconstruction rates

This study is not designed to address any oncology endpoints. The study investigators plan to perform a subsequent randomised-controlled study to evaluate the impact of pre-mastectomy radiotherapy on local control with links to translational research.

**4. Study Design**
A two-centre non-randomised intervention trial investigating whether reversing the order of mastectomy (+axillary nodal clearance) with immediate DIEP flap reconstruction and adjuvant radiotherapy is safe.

The proposed trial intervention will be conducted in compliance with the trial protocol, standard operating procedures, local R&D management guidance, Good Clinical Practice including the Research Governance Framework 2005 (2nd edition) and other applicable regulatory requirement(s) including but not limited to the Human Tissue Act 2004, Human Tissue (Quality and Safety for Human Application) Regulations 2007, the Medical Devices Regulations 2002, and Ionising Radiation (Medical Exposures) Regulations 2000.

**5. Endpoints**

**Primary Endpoint**

- Presence of open breast wound at 4 weeks after mastectomy & DIEP flap reconstruction
  (Open wound defined as wound requiring a dressing />1cm)

**Secondary Endpoints**

- Presence of an open breast wound at 8 and 12 weeks after mastectomy & DIEP flap reconstruction
- Relationship between pre- and intra-operative factors and likelihood of open wound at 4 weeks
- DIEP flap loss rate
- Difference in volume and symmetry between the reconstructed and non-reconstructed breast using 3D-surface imaging at 3 months and 12 months after surgery.
- Patient satisfaction (as measured using the BREAST-Q reconstruction module – see Appendix F) before, three months after, and 12 months after surgery.
- Difference in breast compressibility between the reconstructed and non-reconstructed breast using applanation tonometry at 3 months and 12 months following surgery.

**6. Inclusion / Exclusion Criteria**
Number of patients= 20 total

**6.1 Inclusion criteria**

- Women >18 years with histopathologically-confirmed breast cancer, who:
- require mastectomy for any reason (e.g., extensive disease, failed conservative management etc.)
- axillary nodal clearance
- adjuvant radiotherapy and who are suitable for DIEP flap reconstruction at the time of mastectomy

**6.2 Exclusion criteria**

- Inability to give informed consent
- MDM unable to make recommendation for radiotherapy based on pre-operative histopathological and imaging findings i.e., mastectomy pathology required for MDM to decide on need/ target volume for post-mastectomy RT
- Severe chemotherapy toxicity affecting treatment planning schedule

**6.3 Subject Withdrawal Criteria**

Patients are able to withdraw their consent from the study at any time.

## 7. Methodology

### 7.1 Recruitment

Women with breast cancer who have been recommended by the breast multidisciplinary team to undergo both mastectomy, axillary nodal clearance and adjuvant radiotherapy and who are suitable for DIEP flap reconstruction will be invited to take part in this study at their surgical planning consultation between their 5$^{th}$ and 6$^{th}$ chemotherapy cycles, when it usually becomes apparent, they will require mastectomy and axillary clearance or if they have failed breast conservation. If the patient is interested, they will be given a patient information sheet (see Appendix A) and will be given at least 24 hours to decide whether or not they wish to in participate. If they wish to proceed in the study, they will be invited to a clinical oncology consultation at which time adjuvant radiotherapy will be discussed. If they patient remains happy to participate in the study, consent will be taken prior to radiotherapy and surgery.

### 7.2 Radiotherapy treatment

**Timing**

Radiotherapy will be initiated between 3-4 weeks following the final cycle of chemotherapy.

**Patient positioning and imaging**

All patients will undergo a radiotherapy-planning CT in a standard semi-supine position according to each department's protocol. Where indicated, patients will be imaged using a breath-hold technique (again according to department protocol). Radiotherapy CT scanning should be performed in accordance with department protocol. CT images should be acquired at no greater than 5mm intervals, but ideally at 3mm intervals.

**Target volume definition**

The treatment volume should include the breast +/- supraclavicular fossa +/- internal mammary nodes (IMN) according to departmental policy. The IMN will only be irradiated if there is preoperative involvement on staging CT. The target volume will have been decided at MDT meeting. Target volumes (including breast and regional LN clinical target volumes (CTVs)) should be defined as per ESTRO consensus guidelines (13). CTVs should be expanded by 10mm to produce planning target volumes (PTVs). Heart, ipsilateral and contralateral lung organs-at-risk should be defined as per standard practice.

**Treatment planning**

Treatment plans will be prepared as per standard department practice with the aim of covering the 95% of the breast PTV with the 95% isodose and 80% of the regional nodal PTV with the 95% isodose.

**Dose**

Patients will be treated according to departmental protocol, 40Gy/ 15 fraction/ 3 weeks, 50Gy/25 fractions/5 weeks or 42.72Gy/16#/3.2 weeks. 5mm-10mm wax bolus will be applied to the skin of the breast for half of the planned treatments.

**Treatment verification**

Real-time electronic portal imaging (EPI) will be performed on fractions 1-3 and then weekly as per department protocol.

### 7.3 Surgical treatment

Patients will proceed to mastectomy, axillary nodal clearance and immediate DIEP flap reconstruction at 2-6 weeks following completion of radiotherapy. This range of timings allows for surgery to be planned beyond the peak of the skin reaction but prior to development of skin/ subcutaneous tissue fibrosis.

### 7.4 Follow-up

Patients will be reviewed at 2 (postoperative results clinic – not a data point), 4, 8 and 12 weeks post-surgery (oncoplastic surgery clinics – data point). Patients will undergo 3D-surface imaging*, 2D-photography and applanation tonometry$^{\Psi}$ at 12 weeks and 12 months post-surgery. Patient-reported outcome measures will be assessed at the same time points using the Breast-Q$^{\infty}$(see Appendices D & F). A trial assessing mastectomy and DIEP flap reconstruction (with and without adjuvant radiotherapy), is being assessed in an on-going study at the Royal Marsden by Miss Jenny Rusby (CCR 4283).

*The VECTRA XT 3D surface imaging system (3D-SI) (Canfield Scientific Inc, Fairfield, NJ, USA) is a 3D-photographic image capture system. Six mounted cameras take simultaneous images, which are integrated into a 3D image viewable on a workstation. 3D-SI has been shown to be accurate and reproducible in breast volume calculations (5-7). These accurate volume, symmetry and length calculations allow objective assessment following breast cancer surgery. There are no known harms associated with 3D surface imaging and there is no radiation involved in the imaging acquisition.

∞Various patient-reported outcome measures (PROMs) have been used to evaluate patients' satisfaction after breast cancer treatment (8). The BREAST-Q is a previously validated questionnaire developed quantitatively and qualitatively to measure patients' perceptions before and after breast reconstruction by examining quality of life domains (psychosocial well-being, physical well-being, sexual well-being) and satisfaction domains (satisfaction with breasts, satisfaction with outcome, satisfaction with care) (9,10).

ΨApplanation tonometry is a method of objectively analysing how supple and compressible the breast / reconstructed breast tissue is and was first used in the 1980's to assess breast implant capsular contracture (11,12). A standardized weight balanced applanation disk (305 g, Hillway Applanation Disk; Hillway surgical Ltd., West Sussex, United Kingdom) is used. There are no known harms associated with applanation tonometry.

**8. Data Acquisition**
The following data will be collected for each patient using Case Report Forms (CRFs) (see Appendix C):

**Preoperative**
- Patient demographics: (age, BMI, smoking status, diabetic status)
- Cancer: Date of diagnosis, baseline T-stage, tumour size, grade, LVI, N-stage,
- Treatment: Chemotherapy regimen and date of last cycle, clinical response
- Radiotherapy regimen, start and end dates
- Date of surgery
- Whether or not surgery has been deferred because of RT or chemotherapy toxicity
- Performance status

**Intra-operative**
- Ischaemic time
- Immediate flow: good, acceptable, poor
- Need for anastomotic revision (immediate or return to theatre)
- Operative time
- Blood loss

**Post-operative**
- Drainage volume over first 24hours
- Length of stay (LOS)
- Unplanned return to theatre (and reason) within 24-48hrs, 4 weeks, 8 weeks, + 12 weeks
- DIEP flap failure: total or partial
- Fat necrosis (clinical assessment only)
- Skin envelope necrosis 48hrs, 4 weeks, 8 weeks, and 12 weeks days
- Use of antibiotics for wound related issues
- Number of clinic attendances for wound related problems

**Translational work**
A sample of the tissue (primary breast and nodal) collected for histology will be stored in the Royal Marsden biobank under the study CCR number. This tissue may be used for future ICR translational studies, under the auspices of Professor Kevin Harrington and Dr Navita Somaiah at the Institute of Cancer Research (ICR), providing further information on the genomic changes associated with chest wall radiotherapy.

**9. Data Analysis**

**9.1 Baseline assessments**
Review of clinical records, histopathology reports and imaging to confirm potential eligibility.
Performance status recorded at time of consent.

**9.2 Study assessments**

*From radiotherapy records:*

- Target volume
- Dose
- Use of bolus

*From operation records:*

- Ischaemic time
- Immediate flow: good, acceptable, poor
- Need for anastomotic revision (immediate or return to theatre)
- Operative time
- Blood loss

**9.3 Follow-up assessments**

- Drainage volume over first 24hours
- Length of stay (LOS)
- Unplanned return to theatre (and reason) within 24-48hrs, 30, 60, 90 days
- DIEP flap failure: total or partial
- Fat necrosis (clinical assessment only)
- Skin envelope necrosis 48hrs, 30, 60, 90 days
- Use of antibiotics for wound related issues
- Number of clinic attendances for wound related problems
- Breast-Q score, cosmesis (scored on 2D and 3D surface imaging) and breast compressibility at 12 weeks and 12 months post-surgery

## 10. Study Organisation / Trial Monitoring and Management Strategy

**10.1 Responsibilities**

Principal Investigator: Ms Fiona MacNeill

| Co-investigators: | *RM* | *Imperial* |
|---|---|---|
| Clinical Oncology | Dr Anna Kirby | Dr Suzy Cleator (local PI) |
| | Dr Gillian Ross | Dr Charles Lowdell |
| Breast Surgery | Mr William Allum | Mr Dimitri Hadjiminas |
| | Mr Peter Barry | Mr Ragheed Al-Mufti |
| | Mr Gerald Gui | Mrs Katy Hogben |
| | Miss Rachel O'Connell (Res Fellow) | Mrs Jackie Lewis |
| | Miss Caroline Richardson | |
| | Mrs Nicky Roche | |
| | Ms Jenny Rusby | |
| | Mr Paul Thiruchelvam (SpR) | |
| Plastic Surgery | Mr Stuart James | Mr Simon Wood |
| | Mr Kelvin Ramsey | Mr Navid Jallali |
| | Mr Paul Harris | |
| | Mr Kieran Power | |
| | Mrs Mary Morgan | |

Ms Fiona MacNeill has overall responsibility for running the study and dealing with trial management, in accordance with Research Governance Framework Guidelines, Good Clinical Practice (GCP) and other relevant regulatory requirements. She will have overall responsibility for patient recruitment and consent, day-to-day running of the study, data collection, completion of Case Report Forms, data analysis, and data storage.

Mr Paul Thiruchelvam will undertake data collection, analysis and storage under the supervision of the PI.

*Participating centre (Imperial):*

Participating centres will undertake patient recruitment/ consent, day-to-day running of the study and completion of Case Report Forms (see Appendix C). In addition, the participating centres are required to:

- Put and keep in place arrangements to adhere to the principles of GCP
- Keep a copy of all 'essential documents' (as defined under the principles of GCP) and ensuring appropriate archiving and destruction of documentation once the trial has ended
- Take appropriate urgent safety measures

Risk assessment will be carried out prior to commencement of the study. Adverse events will be reported as outlined in section 12. The trial co-investigators will meet on a monthly basis to monitor progress of the study, with interim meetings as required to discuss any safety issues relating to the study.

### 10.2 Study Procedures

*Protocol compliance:*
PRADA is being conducted in accordance with the professional and regulatory standards required for non-commercial research in the NHS under the Research Governance Framework for Health and Social Care and the principles of GCP. Before activating the trial, participating centres are required to sign an agreement between an individual participating centre and The Royal Marsden NHS Foundation Trust. Centres may commence recruitment once centre agreements have been signed by both parties, trial documentation is in place and a site initiation visit has taken place.

*Protocol amendments:*
Proposed protocol amendments will be submitted to CCR by the Principal Investigator. CCR will agree protocol amendments prior to acceptance and submission to the Main REC. Once approved the Principal Investigator at each centre will be informed of the change and sent all the associated documentation. It is the Principal Investigator's responsibility to submit amendments to their R&D department for approval. Confirmation that this has been done must be provided to CCR.

*Data acquisition:*
Data will be recorded on the PRADA case report forms (CRFs). These will be collated by Mr Paul Thiruchelvam.

By participating in PRADA, the Principal Investigator at Imperial is confirming agreement with his/her local NHS Trust to ensure that:

- sufficient data is recorded for all participating patients to enable accurate linkage between hospital records and CRFs
- source data and all trial related documentation are accurate, complete, maintained and accessible for monitoring and audit visits
- original consent forms are dated and signed by both patient and investigator and are kept together in a central log together with a copy of the specific patient information sheet(s) given at the time of consent
- all essential documents must be retained after the trial ends to comply with current legislation
- staff will comply with the PRADA protocol

*Central Data Monitoring*
Mr Thiruchelvam will review incoming CRFs for compliance with the protocol, and for inconsistent or missing data. Should any missing data or data anomalies be found, queries will be sent to the relevant centre for resolution. Following initial review, the CRF data items will be entered into the clinical study database held at the Royal Marsden NHS Foundation Trust. Data will be further reviewed for data anomalies / missing data. Any systematic inconsistencies identified may trigger monitoring visits to centres.

- Start date definition: The planned start date is 1st September 2015. The PI is aware that she will be notified in writing of CCR / R&D Approval once ethical approval has been given. The local PI is aware that the actual start date will be the date upon which the study is activated by their local R&D department.

- Patient Screening: Patients will be screened as potentially eligible via local breast unit multidisciplinary meetings. Study consent will be taken at the time of consent to radiotherapy.

- Patient Withdrawal: Patients may withdraw from the study at any time. This will not affect the standard of care that they receive.

- Study Completion: Patients will have completed the study twelve months after completion of surgery.

- End of Trial Definition: This is defined as the last patient visit.

## 11. Evaluation of Outcome

**Primary Endpoint**

- Presence of open breast wound at 4 weeks after mastectomy & DIEP flap reconstruction
  (Open wound defined as wound requiring a dressing />1cm)
  - The proportion of patients with an open breast wound at 4 weeks (denominator = number of patients recruited into study).

**Secondary Endpoints**

- Presence of an open breast wound at 8 and 12 weeks after mastectomy & DIEP flap reconstruction
  - The proportion of patients with an open breast wound at 8 and 12 weeks (denominator = number of patients recruited into study).
- Relationship between pre- and intra-operative factors and likelihood of open wound at 4 weeks
- Loss of DIEP flap
- Difference in volume and symmetry between the reconstructed and non-reconstructed breast using 3D-surface imaging at 3 months and 12 months after surgery.
  - For the 20 patients, mean / median (depending on distribution of data) change in volume of the reconstructed breast between 3 and 12 months.
  - For the 20 patients, mean / median (depending on distribution of data) change in symmetry score (reconstructed and contralateral breast) between 3 and 12 months.
- Patient satisfaction (as measured using the BREAST-Q reconstruction module) before, three months after, and 12 months after surgery.
  - Mean/median (depending if normally distributed or not) BREAST-Q score at each time point will be compared using the t test or Mann Whitney U test.
- Difference in breast compressibility between the reconstructed and non-reconstructed breast using applanation tonometry at 3 months and 12 months following surgery.
  - Mean/median (depending if normally distributed or not) applanation tonometry index will be calculated at 3 and 12 months and compared using the t test or Mann Whitney U test.

## 12. Adverse Events

### 12.1 Definitions

*Adverse event:* Any untoward medical occurrence in a patient or clinical trial subject administered a research procedure; events do not necessarily have a causal relationship with the procedure.

*Related adverse event:* An adverse event assessed by the PI or CI as reasonably likely to be related to the administration of a research procedure.

*Serious adverse event:* An untoward occurrence that results in death, is life-threatening, requires hospitalisation or prolongation of existing hospitalisation, results in persistent or significant disability or incapacity, leads to a congenital anomaly or birth defect, or is otherwise considered medically significant by the PI.

### 12.2 Adverse event reporting

All adverse events should be reported in accordance with ICH Good Clinical Practice.

## 13. Statistical Considerations and Pathology Support

### 13.1 Statistical design

A two-centre non-randomised intervention trial investigating whether reversing the order of mastectomy with immediate DIEP flap reconstruction and adjuvant radiotherapy is safe. Statistical support has been provided by Komel Khabra at the RDSU.

#### 13.1.1 Sample size

20 patients.
Pragmatic to allow data collection within a meaningful time frame and based on predicted patient eligibility/ recruitment
(NB In 2013-2014 RMH performed 12 post NACT, mastectomy/DIEP followed by RT).

### 13.2 Statistical Analysis

Descriptive statistics will summarise baseline characteristics. For quantitative data, the mean, median, standard deviations and range will be presented. For qualitative data, proportions and frequencies will be presented with any appropriate 95% confidence intervals.

**Primary endpoint**

This will be presented as the proportion of patients with the presence of open breast wound at 4, 8, 12 weeks (with denominator being the number of patients assessed at each time point).

NB: Analysis of flap loss rates will be continuous as a patient safety measure. Flap loss rates are very low (<1%) in standard practice such that if there are more than two flap losses in the first ten patients the PRADA working group will convene a meeting to discuss stopping the study.

**Secondary endpoints**

- Presence of an open breast wound at 8 and 12 weeks after mastectomy & DIEP flap reconstruction
    - The proportion of patients with an open breast wound at 8 and 12 weeks (denominator = number of patients recruited into study).
- Relationship between pre- and intra-operative factors and likelihood of open wound at 4 weeks
- Loss of DIEP flap
- Difference in volume and symmetry between the reconstructed and non-reconstructed breast using 3D-surface imaging at 3 months and 12 months after surgery.
    - For the 20 patients, mean / median (depending on distribution of data) change in volume of the reconstructed breast between 3 and 12 months
    - For the 20 patients, mean / median (depending on distribution of data) change in symmetry score (reconstructed and contralateral breast) between 3 and 12 mths
- Patient satisfaction (as measured using the BREAST-Q reconstruction module) before, three months after, and 12 months after surgery.
    - Mean/median (depending if normally distributed or not) BREAST-Q score at each time point will be compared using the t test or Mann Whitney U test
- Difference in breast compressibility between the reconstructed and non-reconstructed breast using applanation tonometry at 3 months and 12 months following surgery.
    - Mean/median (depending if normally distributed or not) applanation tonometry index will be calculated at 3 and 12 months and compared using the t test or Mann Whitney U test.

### 13.3. Interim Analyses

No interim analyses are planned.

### 13.4 Pathology Support

Dr Peter Osin and Dr Ashutosh Nerurkar will provide pathology support for the study

## 14. Patient and Public Involvement (PPI)

The Patient Information Sheet (PIS) and Informed Consent Form (ICF) have been reviewed primarily at the Patient Carer Advisory Group for the trust (see Appendices A & B). The documents were presented at a live meeting and collated feedback has subsequently been received, some document changes have been made in response to this to make the PIS more patient friendly.

The PIS and ICF have had a broader review at the Royal Marsden trust's Patient and Carer Research Review Panel (PCRRP) which is a research specific PPI review board. The PCRRP consists of approximately 30 members who are trained in providing research specific review. The documents have received a favourable review from the panel with the following changes made in response to the panel's comments. Study panel contact Mrs Sarah Stapleton sarah.stapleton@rmh.nhs.uk.

## 15. Regulatory & Ethics Committee Approval

### 15.1. Ethical Considerations

The responsible investigator will ensure that this study is conducted in agreement with the Declaration of Helsinki (1964).

The protocol has been written, and the study will be conducted, according to the ICH Harmonized Tripartite Guideline for Good Clinical Practice (ref: http://www.ifpma.org/pdfifpma/e6.pdf).

An application to the main Research Ethics Committee will be submitted once preliminary approval from CCR is given, and approval will be required prior to patient recruitment. The study will be conducted in accordance with the conditions of ethical approval.

The Principal Investigator at each centre is responsible for gaining Site Specific Assessment and Research and Development approval for this study, before entering patients.

**15.2. Informed Consent**

It is the responsibility of the Principal Investigator to give each patient, prior to inclusion in the trial, full and adequate verbal and written information regarding the objective and procedures of the trial and the possible risks involved. Sufficient time (a minimum of 24 hours) should be allowed for the patient to decide on trial entry. Patients must be informed about their right to withdraw from the trial at any time. Written patient information must be given to each patient before enrolment. The written patient information is an approved patient information sheet (PIS) according to national guidelines.

All consent forms must be countersigned by the Principal Investigator or a designated individual. A record listing the designated individuals and the circumstances under which they may countersign consent forms must be clearly documented at the centre as part of the Delegation of Responsibilities Log. This log, together with original copies of all signed patient consent forms, must be available for inspection.

**15.3 Patient Confidentiality**

Patient confidentiality will be maintained at all times. Patients will be asked to consent to their full name, date of birth and hospital number being collected at enrolment in the study.

The principal investigator must keep a log of patients' trial numbers, names, and hospital numbers. The principal investigator must maintain in strict confidence trial documents, which are to be held in the local centre (e.g. patients' written consent forms). The principal investigator must ensure the patient's confidentiality is maintained.

The PRADA Working Group will maintain the confidentiality of all patients and will not reproduce or disclose any information by which patients could be identified. Representatives of the PRADA Working Group and the Radiotherapy QA team will be required to have access to patients notes for quality assurance purposes, but patients should be reassured that their confidentiality will be respected at all times. In the case of special problems, it is also necessary to have access to the complete study records provided that patient confidentiality is protected.

All staff involved in the study will be required to abide by the Data Protection Act 1998.

**16. Data Handling and Record Keeping**

The investigators will make source data and documents available for the purposes of trial-related monitoring, audits, IRB/IEC review and regulatory inspections as required, without compromising patient confidentiality as stated above.

The chief investigator carries overall responsibility for ensuring that data handling and record keeping for this study is in accordance with the Data Protection Act and Caldicott principles. Only the named investigators will have access to study data. Patients will be identified in the trial database according to a unique study number.

CRFs / Surgeons narratives will be stored in a locked cabinet in Miss MacNeill's secure office at RMH. The RMH database will be located as an excel spreadsheet in the restricted access 'BREAST' folder on the Q Drive in the Royal Marsden server. Data will be transferred between sites on an Iron key or via nhs.net.

**17. Financing, Indemnity & Insurance**

The PRADA Study has no formal funding but will be carried out under the 'usual' research funding avenues using the in house research resources.

Indemnity for participating hospitals is provided by the usual NHS indemnity arrangements.

There is collaboration between the Royal Marsden NHS Foundation Trust and the Institute of Cancer Research.

Where the Royal Marsden NHS Foundation Trust is either sponsoring or collaborating with externally sponsored research the NHS Litigation Authority will cover standard clinical negligence by employees, staff and

health professionals employed by the Royal Marsden NHS Foundation Trust. For more information visit the following website:
www.clinical-medical-negligence-injuries.co.uk
There is unlimited liability and no excess. Insurance is provided under the Clinical Negligence Scheme for Trusts and there is no cover for non-negligence claims.
For all notification of claims please contact the Board Secretariat.

**18. Publication Policy**

All presentations and publications pertaining to this study require authorisation from the Chief Investigator, who is responsible for the intellectual property arising from this study.

The Chief Investigator will review submissions for publication. All participating centres and clinicians will be acknowledged in the main trial publication. Authorship of any secondary publications will reflect the intellectual and time input into these studies and will not be the same as on the primary publication. Any publications or presentations relating to this study will be submitted in accordance with CCR policy and must also be submitted to the NIHR for approval. No investigator may present or attempt to publish data relating to the PRADA study without prior permission from the PRADA Working Group.

**19. Abbreviations**

| | |
|---|---|
| BC | Breast cancer |
| CRF | Case report form |
| DVH | Dose-volume histograms |
| GCP | Good Clinical Practice |
| IMRT | Intensity-modulated radiotherapy |
| LAD | Left anterior descending coronary artery |
| NCRI | National Cancer Research Institute |
| PIS | Patient information sheet |
| RT | Radiotherapy |
| v_DIBH | Voluntary deep-inspiratory breath-hold |
| PIS | Patient Information Sheet |
| ICF | Informed Consent Form |
| PCRRP | Patient and Carer Research Review Panel |
| CTVs | Clinical target volumes |
| PTVs | Planning target volumes |

**20. Protocol References**


1. Rastogi P et al. Preoperative chemotherapy: updates of National Surgical Adjuvant Breast and Bowel Project Protocols B-18 and B-27. JCO, 2008, 10;778-85
2. EBCTCG. Effect of radiotherapy after mastectomy and axillary surgery on 10-year recurrence and 20-year breast cancer mortality: meta-analysis of individual patient data for 8135 women in 22 randomized trials. Lancet, 2014, 383:2127-35
3. Sebag-Montefiore D et al. Pre-operative radiotherapy versus selective postoperative chemo-radiotherapy in patients with rectal cancer (MRC CRC-07 and NCIC-CTG C016): a multicentre randomised trial. Lancet, 2009, 7;811-20
4. Tansley P et al. New treatment sequence protocol to reconstruct locally advanced breast cancer. ANZ J Surg, 2013, 83;630-5
5. Yip JM, Mouratova N, Jeffery RM *et al.* Accurate assessment of breast volume: a study comparing the volumetric gold standard (direct water displacement measurement of mastectomy specimen) with a 3D laser scanning technique. *Ann Plast Surg* 2012; 68(2):135-41.
6. Henseler H, Khambay BS, Bowman A *et al.* Investigation into accuracy and reproducibility of a 3D breast imaging system using multiple stereo cameras. *J Plast Reconstr Aesthet Surg* 2011; 64(5):577-82.
7. Kovacs L, Eder M, Hollweck R *et al.* Comparison between breast volume measurement using 3D surface imaging and classical techniques. *Breast* 2007; 16(2):137-45.
8. Kanatas A, Velikova G, Roe B *et al.* Patient-reported outcomes in breast oncology: a review of validated outcome instruments. *Tumori* 2012; 98(6):678-88.
9. Cano SJ, Klassen A, Pusic AL. The science behind quality-of-life measurement: a primer for plastic surgeons. *Plast Reconstr Surg* 2009; 123(3):98e-106e.
10. Cano SJ, Klassen AF, Scott AM, Pusic AL. A closer look at the BREAST-Q((c)). *Clin Plast Surg* 2013; 40(2):287-96.

11. Moore JR. Applanation tonometry of breasts. *Plast Reconstr Surg* 1979; 63(1):9-12.
12. Gylbert LO. Applanation tonometry for the evaluation of breast compressibility. *Scand J Plast Reconstr Surg Hand Surg* 1989; 23(3):223-9.
13. Offersen et al, ESTRO consensus guideline on target volume delineation for elective radiation therapy of early stage breast cancer. Radiother Oncol 2015; 114(1):3-10.

Protocol **Appendices**

A - Patient information sheet (PIS)_RMH_V2
B - Consent form_RMH_V2
C - CRF PRADA Study_v1
D - Qualitative (3D Photography/Applanation) CRF_RMH_v1
E - BREAST-Q CRF_RMH_v1
F - BREAST Q-ReconstructiveModule-Questionnaire

## PRADA Trial Management Group Author List

| First name, initials | Surname |
|---|---|
| | |
| Paul TR | Thiruchelvam |
| Daniel R | Leff |
| Amy R | Godden |
| Susan | Cleator |
| Simon H | Wood |
| Anna M | Kirby |
| Navita | Somaiah |
| Neill | Patani |
| Dorothy | Gujral |
| Gillian | Ross |
| Stuart | James |
| Aadil | Khan |
| Jennifer E | Rusby |
| Dimitri | Hadjiminas |
| Fiona A | MacNeill |
